# Tracing the natural course of visual acuity and quality of life in neovascular age-related macular degeneration: a systematic review and quality of life study

**DOI:** 10.1186/s12886-017-0514-3

**Published:** 2017-07-11

**Authors:** Mari Elshout, Carroll A. Webers, Margriet I. van der Reis, Yvonne de Jong-Hesse, Jan S. Schouten

**Affiliations:** 1 Maastricht University Medical Center, University Eye Clinic Maastricht, PO Box 5800, 6202 AZ Maastricht, The Netherlands; 2 Department of Ophthalmology, VU University Medical Center, Amsterdam, The Netherlands

**Keywords:** Age-related macular degeneration, Natural progression, Visual acuity, Quality of life, Prognosis, Cost-effectiveness

## Abstract

**Background:**

Describing the natural course of neovascular age-related macular degeneration (nAMD) is essential in discussing prognosis and treatment options with patients and to support cost-effectiveness studies.

**Methods:**

First, we performed a literature search in PubMed, Embase, and Cochrane. We included randomized clinical trials and prospective observational studies reporting visual acuity (VA) in non-treated patients, 24 studies in total. We integrated VA data using best fit on Lineweaver-Burke plots and modelled with non-linear regression using reciprocal terms. Second, we performed a quality-of-life (QoL) study in nAMD patients. We measured VA with Radner reading charts and QoL with the Health Utilities Index issue 3 (HUI-3) questionnaire in 184 participants. We studied the relation VA-QoL with linear regression. Third, with Monte Carlo simulation, we integrated the VA model from the literature review and the relation VA-QoL from the QoL study.

**Results:**

Visual acuity was 0.4 and 0.07 after 5 years in the better-seeing, and worse-seeing eye, respectively. After 4.3 years, VA was <0.5 in the better-seeing eye; <0.3 after 7 years; 0.05 after 17 years. QoL score decreased from 0.6 to 0.45 after 10 years.

**Conclusions:**

The natural course of nAMD in both eyes needs to be considered when informing patients. Visual acuity in the best eye decreases to below 0.5 in 4.3 years. This affects QoL significantly.

**Electronic supplementary material:**

The online version of this article (doi:10.1186/s12886-017-0514-3) contains supplementary material, which is available to authorized users.

## Background

Neovascular age-related macular degeneration (nAMD) is a common disorder of the ageing eye. Left untreated, it leads to severe central visual impairment.

“Will I become blind?” is a question patients with nAMD commonly have. Blindness is feared among patients [1]. Legal blindness of the affected eye is inevitable when nAMD is left untreated. Answering questions regarding the outcome of nAMD requires making predictions. Predicting prognosis is one of the most challenging aspects of clinical medicine, for several reasons. First, a degree of uncertainty exists in results presented in scientific literature. Second, uncertainty is involved when estimating prognosis for the individual patient. Third, nAMD is a bilateral disease. Binocular legal blindness and severe loss of quality of life (QoL) will not occur until the fellow eye becomes significantly affected by nAMD. As QoL greatly depends on visual acuity (VA) in the better-seeing eye, VA in each of both eyes needs to be taken into account when predicting outcomes.

New treatments have become available for nAMD, along with concerns regarding the associated utilisation of resources. In this context, cost-effectiveness studies have been published on the subject of treating nAMD [2]. In performing a cost-effectiveness study, a good estimate of the natural progression of nAMD is useful for modelling a comparator treatment such as ‘best supportive care’ without an active treatment, or the course of the disease after discontinuation of treatment [3]. As trials with no-treatment arms are no longer being conducted, estimates of VA and QoL in non-treated eyes have to be based on existing data. Basing assumptions on only one or a few studies may render the results less precise than basing them on results of a systematic review.

In the systematic review section of this study, the question being addressed is what the VA loss is over time in eyes affected with nAMD. Therefore, we selected studies involving patients with nAMD and with the neovascular lesion specified as occult, minimally classic or predominantly classic; not receiving an active intervention; and with VA as an outcome. Study designs could be retrospective studies, prospective clinical studies or observational cohort studies. In the QoL study section of this study, we address the correlation of VA and QoL in order to translate VA results from our review to QoL scores. In this article we describe the long-term natural course of VA and QoL in each eye of patients with nAMD. The results will help the clinician to inform patients about their future VA and to discuss treatment options. Furthermore, the results can be used in cost-effectiveness analysis when comparing a proposed treatment to a ‘no treatment’ or ‘best supportive care’ alternative.

## Methods

### Visual acuity: literature search and meta-analysis

We collected and integrated VA data in a systematic literature review with meta-analysis. We searched for articles in the Databases PubMed; Embase; and the Cochrane databases on Systematic Reviews and Clinical Trials with the last search on 20 January 2015 with no date limits applied. We used the following search terms in the databases: (Macula* OR AMD) AND (follow-up OR longitudinal OR outcome OR randomised OR epidemiologic*) AND (vision OR visual acuity) AND (placebo OR sham OR natural OR no-treatment OR untreated).

One author (ME) was involved in the literature selection process. We included published studies that included non-treated or placebo-treated nAMD patient groups. Studies needed to report separate baseline and follow-up VA outcomes for occult (OC), minimally classic (MC) and/or predominantly classic (PC) lesions. Follow-up period was one year at minimum. Exclusion criteria were: disease studied other than nAMD; publication types such as reviews and conference abstracts; VA not reported as outcome; only treated patients described; the lesion types not being specified; Languages used other than Dutch; English; French; German and Spanish. No additional public protocols are available. We assessed titles and abstracts of the articles returned by the search and excluded articles that were not of relevance. The remaining articles were in- or excluded after reading and assessing the full-text.

Data sought and recorded were neovascular lesion type; baseline VA; follow-up VA values and the corresponding follow-up time points. We performed data extraction on the principal summary measure (VA & time-point) by recording all VA data from the report with the corresponding time-point of measuring, copying directly from the full-text or tables; and digitally measuring VA and time-point in figures. Risk of bias was not assessed, as we studied only non-treatment arms of included studies.

We integrated VA data using the method of Shah et al. [4, 5]. This is a published method which offers solutions for several concerns in combining data regarding VA loss in nAMD: The difference in average baseline VA among studies; the non-linear progression of VA loss; and the variety of the course of VA between studies. The method is based on the assumption that the difference in baseline VA, between studies and between lesion types, arises from the difference in time of inclusion of patients in the studies. Further, it assumes the progression of VA to be non-linear over time. Rather, it assumes that VA loss follows a reciprocal course, which fits the perception in nAMD that VA loss is more severe immediately after the occurrence of exudative disease, and tends to plateau to an end-stage VA level.

The method involves the following steps: First, we Integrated VA data on double reciprocal (Lineweaver-Burke) plots. Here, the x-axis represented 1/(*Months After Enrolment*). The y-axis represented 1/(*Letters Lost)*, where *Letters Lost* = 85 – *Study VA*, in Early Treatment Diabetic Retinopathy Study (ETDRS) Letters. We combined the data of all included studies in one plot. We also combined VA data for each lesion type separately on three more plots.

Second, we added a horizontal translation factor, to the initial time values of each individual data set, expressed in months. This changed the horizontal axis from 1/(*Months After Enrolment*) to 1/(*Months of Exudative Disease*) where *Months of Exudative Disease* = *Months After Enrolment* + *Horizontal Translation Factor*. In the previous studies by Shah et al., to determine the optimum horizontal translation factor for each data subset, an arbitrary horizontal translation factor to each data subset was added until the correlation coefficient *r* ^*2*^ for the overall linear trend line was maximized. This was done in an iterative fashion until there was no further change in *r* ^*2*^ as additional translations were introduced. Given the number of studies to be included in our study, a manual iterative method seemed neither practical nor precise. We employed a Macro-operated Excel spreadsheet to add arbitrary horizontal translation factors to each subset in an automated iterative fashion. The analysis comprised 1,000,000 iterations in total, from which we selected the set of horizontal translations producing the best linear fit (highest *r* ^2^). Consecutively, we could express VA as a function of ‘Months of Exudative Disease’, with non-linear regression using reciprocal terms. The general formula of such a double reciprocal model is as follows:

$$ \frac{1}{letters\kern0.5em  lost}= a+\frac{b}{t} $$

Here, *t* = *Months of Exudative Disease*. End-stage VA loss approaches 1/*a*. With higher *a*, end-stage VA is better [5]. With lower *b*, the progression toward end-stage VA loss is more rapid.

### Quality-of-life study

We undertook a cross-sectional study to measure QoL in nAMD patients. We included patients from the University Eye Clinic Maastricht; the Catharina Hospital Eindhoven; the VU University Medical Center in Amsterdam, and ZorgSaam Hospital Zeeuws-Vlaanderen, in The Netherlands. The Medical Ethical Committees of all centres approved the design of the study according to Dutch law and the Declaration of Helsinki. Patients were aged at least 50 years and diagnosed with nAMD with choroidal neovascularisation (CNV) on fluorescein angiography between 1992 and 2011. We screened 1948 files; 588 patients were eligible. One hundred and eighty-four patients agreed to participate. Patients gave written informed consent before taking part.

Two authors (ME and MvdR) visited the patients at home between March 2010 and November 2011. We measured VA using Radner reading cards [6], and by assigning counting fingers or seeing hand motions the appropriate value on the Snellen scale. We converted VA results to ETDRS letters equivalents. We measured QoL using the Health Utility Index issue 3 (HUI-3) questionnaire [7]. We performed linear regression analysis with the HUI-3 score as dependent; VA in the best eye (ETDRS) as independent variable. Further details on the QoL study can be found in the Additional file 1. Other results of this study have been published previously [8].

### Integrating visual acuity and quality-of-life

We traced the natural progression of VA in nAMD, with the corresponding deterioration in QoL. We used a Monte Carlo simulation developed for the progression of non-treated and treated eyes with nAMD [9]. The technical structure of the nAMD model was based on an earlier model for studying the progression of glaucoma [10]. The simulation was developed in Microsoft Excel. In the simulation, virtual patients (‘entities’) are simulated one by one. The simulation traces the course of VA in both, treated and non-treated, eyes. In the simulation for this study, both eyes remained non-treated. At baseline, both eyes have good VA, and at least one eye is affected. A number of parameters in the model define the longitudinal behaviour of VA and QoL in the simulation. First, VA in affected eyes is defined by the reciprocal terms from the regression analysis on the combined literature analysis described earlier in this paper. Second, the conversion of non-affected eyes to affected eyes is based on a hazard rate, which is calculated using the incidence of nAMD in fellow eyes, based on an earlier review [11]. Third, QoL was calculated using the regression coefficients from the QoL study described earlier in this paper. One thousand entities were simulated and the average loss of VA and QoL in each eye could be traced. Further details on the Monte Carlo simulation and the parameters used can be found in the Additional file 1.

## Results

### Review: visual acuity

**Fig. 1:**
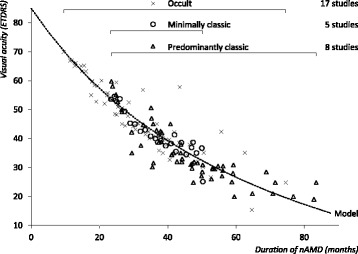
Visual acuity in eyes with non-treated neovascular age-related macular degeneration. Combined analysis. *nAMD* neovascular age-related macular degeneration

**Fig. 2:**
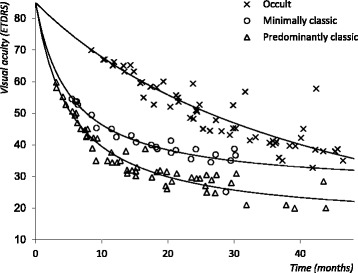
Visual acuity in eyes with neovascular age-related macular degeneration. Separate analysis per lesion type

The literature search returned a total 1385 articles. All articles were assessed for eligibility. The number of articles excluded based on title and abstract screen was 218. We included 24 articles in the review, totalling 2293 affected, non-treated eyes. Table 1 displays a summary of the studies. Table 1 also lists the horizontal translation factors added to the original data to achieve the best linear fit on the double reciprocal scale. Figure 1 shows the results of the Shah [5] analysis, all included studies combined. Figure 2 shows the results of the analysis separated per lesion type. The Shah analysis produces essentially a non-linear analysis with reciprocal terms *a* and *b*. These terms are specified in Table 2. End-stage VA loss approaches 1/*a*. With a higher *a*, the end-stage VA is better. With a lower *b*, the progression toward the end-stage VA loss is more rapid. The model defined by the reciprocal terms from analysis that includes all studies and lesion types is used in the Monte Carlo simulation to calculate VA over time. The total loss in VA over 24 months is greatest in PC lesions, least in OC lesions, and intermediate in MC lesions.

**Table 1:** Studies describing visual acuity loss secondary to neovascular age-related macular degeneration

Study	Design	Follow-up (months)	Eyes	Baseline VA (ETDRS)	Horizontal translation factor (months)	
					Lesion-specific analysis	Combined analysis
Occult lesions						
Arnold [15]	RCT	38	29	60.3	15.85	17.7
Blinder - TAP & VIP studies [16]	RCT	24	92	65.2	14.38	15.5
Boyer - MARINA study [17]	RCT	24	151	53.6	21.63	23.0
Bressler [18]	OCS	36	84	55	21.52	22.3
Bressler - MPS study [19]	RS	60	26	65	13.31	14.6
Cardillo-Piccolino [20]	OCS	20	63	65.1	11.70	12.8
Eter [21]	RCT	6	15	58.2	18.32	19.5
Gragoudas - VISION study [22]	RCT	12	120	52.7	24.77	25.4
Gustavsson [23]	RCT	12	9	45	40.46	36.9
Jaakkola [24]	RCT	36	45	52.5	29.87	28.6
Kaiser - VIO study [25]	RCT	24	120	55.7	21.26	22.4
Kobayashi [26]	RCT	24	7	59.9	16.12	17.9
Krebs [27]	OCS	3	107	67	10.46	11.9
Myint [28]	RCT	12	11	50.5	24.38	26.6
Pece [29]	OCS	13.5	383	66.9	10.29	11.5
Soubrane [30]	RS	34	82	69.9	8.42	9.5
Tholen [31]	RCT	24	21	66.2	11.86	13.1
Minimally classic lesions						
Bressler - VIM study [32]	RCT	24	40	54.5	5.49	24.42
Blinder - TAP & VIP studies [16]	RCT	24	104	53.7	6.22	23.43
Gragoudas - VISION study [22]	RCT	12	102	52.7	6.41	25.10
Schneider [33]	RS	8.3	4	41.3	20.74	41.99
Boyer - MARINA study [17]	RCT	24	87	53.6	6.04	23.43
Predominantly classic lesions						
D’Amico - AA study [34, 35]	RCT	24	30	47	6.35	35.24
Blinder - TAP & VIP studies [16]	RCT	24	83	50.6	5.60	35.14
Bressler - MPS study [19]	RS	60	119	59.7	3.15	23.51
Gragoudas - VISION study [22]	RCT	12	76	52.7	4.59	25.40
MPS study [36]	RS	42	184	45	7.89	40.82
Mandai [37]	RCT	36	4	44.7	7.53	32.85
Valmaggia [38]	RCT	18	29	42.5	7.68	37.90
Behrendt [39]	RCT	12	66	58	3.17	23.85

*ETDRS*, Early Treatment Diabetic Retinopathy Study, *OCS* observational case series, *RCT* randomized controlled trial, *RS* retrospective study, *VA* visual acuity

**Table 2:** Parameters to calculate expected visual acuity loss in neovascular age-related macular degeneration

	Parameter		
Lesion type	*a*	*b*	R^2^
Combined model	0.567	0.0077	0.91
Occult	0.478	0.010	0.90
Minimally classic	0.089	0.017	0.95
Predominantly classic	0.077	0.014	0.96

1/letters lost = *a* + *b* / months

### Quality of life

The QoL study included 184 patients. Average age was 81 years, 52% were female. Participants had an average disease duration of 5.8 years. Visual acuity was 49 letters in the better-seeing eye; 3.5 letters in the worse-seeing eye. Average HUI-3 score was 0.46.

Univariate regression analysis showed the highest correlation (R^2^) of QoL with VA in the better-seeing eye (0.20) and VA in both eyes (0.21) than VA in the worse eye (0.08). Gender and age were not statistically significant predictors of QoL. The presence of comorbidities had a statistically significant impact on QoL, but did not change the correlation parameters of VA in the better-seeing eye in multivariate regression analysis. Because of sufficient correlation with QoL, we used the linear regression coefficient of VA in the better-seeing eye as a factor to calculate QoL in further analysis described below. The linear regression coefficient was 0.004 (*p* 0.000), with a constant of 0.270. This implies a 0.004 decrease in HUI-3 QoL score per letter of VA lost. Further details of the QoL study can be found in the Additional file 1.

### Integrating visual acuity and quality-of-life

**Fig. 3:**
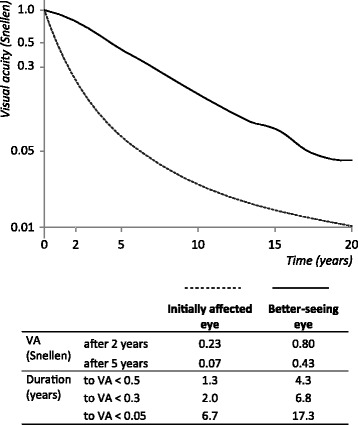
Visual acuity development and benchmarks in neovascular age related macular degeneration. *VA* Visual acuity

The Monte Carlo simulation traced the course of VA over time in both non-treated eyes of nAMD patients, see Fig. 3. The first affected eyes show average VA loss that reflects the double reciprocal model of the literature analysis, shown in Fig. 1. The better-seeing eyes (or ‘fellow eyes’) are mostly unaffected at baseline. A growing proportion of these fellow eyes become affected over time. Therefore, Fig. 1 shows that the better-seeing eyes lose VA more gradually than do the primarily affected eyes. In the Figure, visual acuity can be traced at selected time-points. After five years, VA is 26.7 letters (0.07 Snellen) in the worse-seeing eye, and 66.8 letters (0.43 Snellen) in the better-seeing eye. After ten years, VA is 4.5 letters (0.02 Snellen) in the worse-seeing eye, and 49.8 letters (0.2 Snellen) in the better-seeing eye. Also, the time until certain VA thresholds are reached can be traced. In the better-seeing eye, A Snellen VA of <0.5 (70 ETDRS letters) is reached after an average of 4.3 years. VA < 0.3 (60 letters) is reached after 6.8 years; < 0.05 (20 letters) after 17.3 years.

**Fig. 4:**
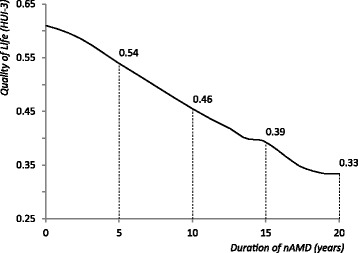
Quality-of-life over time in non-treated neovascular age-related macular degeneration

The simulation also traced QoL over time. Quality-of-life was calculated from better-seeing eye VA using the linear parameters from the QoL study. The QoL values over time are displayed in Fig. 4. Average VA in the better-seeing eye decreases over time. Hence, QoL also decreases over time.

## Discussion

We integrated VA data from non-treated nAMD patients from multiple studies. The included studies differed by the type of CNV lesion studied; the baseline VA of the patients; the duration of follow-up, and the timing of follow-up VA measurements. A fitting solution to these issues was demonstrated in previous studies using double reciprocal plots and adding horizontal translation factors [4, 5]. The review is limited by the fact that study size could not be accounted for in the analysis, which is inherent to the method used. The combined analysis shows that occult lesions are associated with better VA, and are seen more often earlier in the disease course. Later, classic lesions may be seen, with generally worse VA. Initially, VA loss is about 1.25 ETDRS letters per month.

The integrated VA results from the review were combined with QoL data from a cross-sectional QoL study into a Monte Carlo analysis that longitudinally traced VA and QoL in typical patients. Rates of function loss and the corresponding changes in QoL have been studied previously in other eye diseases. For example, a study by Medeiros et al. evaluated the rates of visual field loss and longitudinal changes in QoL in glaucoma patients [12]. In this study, patients received QoL measurements annually and perimetry at six-month intervals, and a significant correlation between QoL score and binocular perimetry sensitivity was demonstrated. Recruitment and follow-up of patients is time- and capital-intensive, and sometimes, when studying the natural course of a disease, may encounter ethical barriers. Computational modelling can solve such issues. Using modelling to translate cross-sectional data into longitudinal outcomes, as in our study, has also been performed previously. Our model was based on a disease-progression model developed and validated for the progression of glaucoma by Van Gestel et al. [10] Similar to our study, QoL on the HUI-3 scale was calculated using the linear coefficients from regression analysis. In the glaucoma model, QoL was based in part on mean deviation on the visual fields, using a cross-sectional QoL study in glaucoma patients. In our study, QoL was calculated using the coefficients from our cross-sectional QoL study in nAMD patients. The glaucoma model also simulated conversion from ocular hypertension to glaucoma, using a hazard rate to generate a distribution of time-to-conversion. In a technically similar approach, our model generated a distribution of time to the development of nAMD in a non-affected eye using a hazard rate from a previous review [11]. The glaucoma model used the rate of mean deviation from a glaucoma population, while we used the rate of loss of VA from the review presented here.

With untreated nAMD, VA decreases, and QoL declines significantly. Visual acuity loss impacts QoL in part through a negative impact on activities of daily living, and the impact on these activities is considerable. As an example, one of the more highly valued activities by individuals is driving. Driving is allowed with VA of > = 0.5 Snellen in a least one eye. Left untreated, nAMD causes a VA loss to below 0.5 within 5 years in the better-seeing eye. Patients may lose about ¼ of their initial QoL in the first 10 years. Quality of life scores for patients with nAMD are comparable to or worse than with several other chronic and/or debilitating diseases, and may decline further over years. Patients with nAMD progress through QoL scores lower than those for example in persons with diabetes (HUI-3 score 0.78), colorectal cancer (HUI-3 score 0.78) [13], or multiple sclerosis (HUI-3 score 0.64) [14]. In this perspective, with effective treatments being available, the clinician should take the highest effort to prevent VA loss in nAMD patients.

The results of this study can be used to inform patients in a more accurate and understandable way about their future sight and visual impairment. Researchers and clinicians can use these results when seeking to estimate the natural progression of nAMD. The disease has a great impact on an individual as well as on a societal level. This study can be used to show how large this impact is or how large it can be. There is increasing discussion regarding the benefits and costs of nAMD and its treatments. Model-based cost-effectiveness studies support this discussion with evidence. In many such studies, a ‘no-treatment’ strategy is used as a comparator strategy. As such, information on natural progression of nAMD has again become highly relevant. The formula and parameters provided can be readily implemented in model-based cost-effectiveness studies.

When explaining prognosis to nAMD patients, it is important to describe the expected progression for both eyes. Especially important is the fellow, and mostly better-seeing, eye. If VA decreases here, binocular VA and QoL are greatly affected. The rate of VA loss in affected eyes is well described in multiple studies [11]. Less is published about VA in the fellow eye or about binocular VA. We know that initially, the fellow eye is mostly unaffected. When nAMD occurs, VA decreases. Our simulation incorporates the incidence of nAMD and the corresponding rate of VA loss in fellow eyes, and predicts outcomes for both eyes.

Will a patient become blind? The World Health Organization (WHO) provides a definition of blindness as Snellen VA being <0.05. Assuming a non-treated 75-year patient with a life expectancy of 11 years, he or she is unlikely to become completely blind. However, it is of paramount importance to consider the serious impact on QoL. With the availability of current anti-vascular endothelial growth factor (anti-VEGF) treatment, this can be presented as a worst-case scenario, e.g. when treatment fails or is not accepted. When informing patients, the benefit of a treatment can be illustrated by comparing treatment outcomes to a hypothetical scenario in which no treatment would be given.

## Conclusions

In conclusion, VA data of the natural course of nAMD from multiple studies were integrated. Visual acuity loss was translated with a 2-eye model into a longitudinal trace of QoL. This provides a comprehensive model of the disease in both eyes, with the patients’ corresponding general well-being, which can be used to support treatment decisions, and to inform patients about their prognosis. Left untreated, the VA will drop to below 0.5 Snellen within 4.3 years.

## Additional file

Additional file 1: Appendix to the paper, providing further details on methodology of the QoL study and Monte Carlo simulation. (PDF 799 kb)

## Abbreviations

anti-VEGF: anti-vascular endothelial growth factor
CNV: Choroidal neovascularisation
ETDRS: Early treatment diabetic retinopathy study
HUI-3: Health utilities index issue 3
MC: Minimally classic lesion
nAMD: Neovascular age-related macular degeneration
OC: Occult lesion
OCS: Observational case series
PC: Predominantly classic lesion
QoL: Quality of life
RCT: Randomized controlled trial
RS: Retrospective study
VA: Visual acuity
WHO: World health organization
