# Supplementary material for: Tracing the natural course of visual acuity and quality of life in neovascular age-related macular degeneration: a systematic review and quality of life study
Source: BMC Ophthalmol. 2017 Jul 11;17:120. doi: 10.1186/s12886-017-0514-3 (PMC5504631; doi:10.1186/s12886-017-0514-3)
Supplement: Additional file 1: — Appendix to the paper, providing further details on methodology of the QoL study and Monte Carlo simulation. (PDF 799 kb) [file 12886_2017_514_MOESM1_ESM.pdf]

APPENDIX

DECEMBER 2016

APPENDIX TO:
TRACING THE NATURAL COURSE OF VISUAL ACUITY AND QUALITY OF LIFE IN NEOVASCULAR AGE-RELATED MACULAR DEGENERATION: A SYSTEMATIC REVIEW AND QUALITY OF LIFE STUDY

MARI ELSHOUT
CARROLL A. WEBERS
MARGRIET I. VAN DER REIS
YVONNE DE JONG-HESSE
JAN S. SCHOUTEN

INTRODUCTION

This document is Appendix to the manuscript “Tracing the natural course of visual acuity and quality of life in neovascular age-related macular degeneration: a systematic review and quality of life study”. In the manuscript, the natural course of neovascular age-related macular degeneration (nAMD) is described. The study comprises three main analyses: (1) a literature review and meta-analysis of the longitudinal course of visual acuity; (2) a study of quality of life (QoL) in patients with nAMD; and (3) combining results of the review and QoL study into a Monte Carlo simulation to analyze the longitudinal course progression of visual acuity and QoL over time. As the manuscript comprises multiple sections of specialized analytics, the reader may need further specifications on the methods and analysis that underpin the analyses. This appendix provides further details on methodology of the QoL study and Monte Carlo simulation.

THE QUALITY-OF-LIFE STUDY

DESIGN AND PARTICIPANTS

The quality-of-life (QoL) study was a cross-sectional, observational study. The study enrolled patients from the University Eye Clinic Maastricht; the Catharina Hospital Eindhoven; ZorgSaam Hospital Zeeuws-Vlaanderen, and the VU University Medical Center in Amsterdam, in The Netherlands. Inclusion criteria were: age of 50 years or more, and a diagnosis with nAMD with choroidal neovascularisation on fluorescein angiography between 1992 and 2011. The number of files screened was 1944. The number of eligible patients was 588. One-hundred and eighty-four patients agreed to participate in the study. Between those patients who participated and those who did not participate, there were no differences in age, gender or geographic location. Questionnaire completion rates exceeded 95% for the HUI-3 measure. Interviews took place between March 2010 and November 2011.

DATA COLLECTION

Visual acuity (VA) and QoL were measured at the participants’ homes. Visual acuity was measured using Radner Reading charts with separate charts for each eye separately and both eyes simultaneously.[1, 2] Counting fingers and seeing hand motions were scored the appropriate values on the decimal visual acuity scale. LogRad and decimal visual acuity were converted to ETDRS visual acuity equivalents to allow for linear regression analysis. The Health Utilities Index Mark III (HUI-3) was used to measure generic quality of life. The HUI-3 is a utility scale, a questionnaire used to assess the participants’ quality of life. Other quality-of-life scales were also applied. The nature of any comorbidities was recorded and scored as on the Charlson Comorbidity Index[3] (CCI), in order to capture the number and the severity of comorbidities in a single metric ranging from 0 to 6.

DATA ANALYSIS AND RESULTS

Descriptive analysis was used to summarize the characteristics of the participants, shown in Table 1.

Table 1.
Characteristics of participants with nAMD.

	**N=184 Mean (SD)**
Age, years	81.2 (7.1)
Female, %	52.2
Time since diagnosis, years	5.8 (3.4)
Charlson Comorbidities Index	0.51 (0.9)
Better-seeing eye VA	
ETDRS	49 (32)
Snellen	0.19
Fellow eye VA	
ETDRS	3.5 (36.5)
Snellen	0.023
Binocular VA	
ETDRS	46 (32)
Snellen	0.17
HUI-3 score	0.45 (0.31)

ETDRS, Early Treatment Diabetic Retinopathy Study;

SD, standard deviation; VA, visual acuity.

Multiple and single linear regression analysis was used to study the relation between VA and QoL. There was a linear trend of better QoL with better VA (Table 2 and Table 3). The highest percentage of explained variability in the single regression analysis (R^2) was observed for better-seeing eye VA (20%) and binocular VA (21%), compared to worse-eye VA (8.6%).

In a multiple regression model (Table 3), the relationship of both binocular VA and comorbidity with utility reached high statistical significance. Variables ‘age’ and ‘living situation’ did not reach significance at the 5% level. Gender; the interaction of visual acuity with comorbidity; and time since diagnosis were not statistically significant predictors of the HUI-3 score in a multiple regression model.

Table 2. Results of single regression analysis for patients with nAMD. Dependent variable: HUI-3 score.

Variable	β	SE	Standardised β	*P*
B_0 (constant)	.270	.035		.000
Best eye VA	.004	.001	.445	.000
R^2 = 0.198				

SE, standard error; VA, visual acuity.

Table 3. Results of multiple regression analysis for patients with nAMD. Dependent variable: HUI-3 score.

Variable	β	SE	Standardised β	*P*
B_0 (constant)	0.666	0.270		0.014
Best eye VA	0.004	0.001	0.429	0.000
Comorbidity (CCI)	-0.104	0.020	-0.321	0.000
Age	-0.006	0.003	-0.129	0.057
Living alone (1) together (2)	0.076	0.040	0.123	0.057
R^2 = 0.352				

CCI, Charlson Comorbidity Index;[3] SE, standard error; VA, visual acuity.

THE MONTE CARLO SIMULATION

The computerized model used to trace the natural course of neovascular age-related macular degeneration is a patient-level, discrete-event, Monte Carlo type simulation. Patient-level Monte Carlo means that virtual patients (called 'entities') are simulated one by one. At baseline, or t=0 in the simulation, the necessary characteristics of the individual entity, such as visual acuity, but also the timing of certain key events, are defined from a set of distributions. Outcomes of each entity are recorded so that results of a population of entities can be interpreted with appropriate statistics. Discrete-event means that the course of time and the re-calculation of entities' parameters model is based on events. Examples of such events are treatment visits, the end of the time-frame. At every event, a set of characteristics of the entity are re-calculated or re-defined, such as disease state; visual acuity; and the time to the next event.

Technically, the model is a Macro-operated spreadsheet developed in Microsoft Excel. For a single entity, the Excel sheet calculates the complete sequencing and timing of all events and all outcomes at each event, until a pre-set time-horizon is reached. The Macro program commands the Excel sheet to record all outputs and repeat the calculations for the next entity, until a population of entities is complete. The virtual population comprised 1,000 entities: Averages and standard deviations of VA and QoL outcomes stabilized at around 500 to 600 entities.

The model has been employed previously to calculate the cost-effectiveness of treatments of macular degeneration.[4] The model was based on a previous model simulating the disease progression of glaucoma.[5] For the current purpose of tracing the natural course of neovascular age-related macular degeneration, the model could be simplified. In the current model, a limited set of parameters eventually define the outcome of interest: the average course of VA and QoL over time in two eyes. These parameters and their distributions are described below in a point-by-point fashion.

BASELINE VISUAL ACUITY

Baseline visual acuity of each eye of an entity at t=0, affected or not, was assumed to be 85 ETDRS letters, or 1.0 Snellen visual acuity.

PROPORTION OF EYES AFFECTED AT BASELINE

An entity had at least one eye affected at baseline: the initially affected eye. The fellow eye could either be affected or not. Each entity had a 5.4% chance of having the fellow eye affected at baseline. This percentage was based on the clinical data from the 184 participants in the cross-sectional study, of which 5.4% had bilateral disease at presentation. We found no other evidence in literature defining the proportion of neovascular AMD patients at presentation with bilateral active disease.

COURSE OF VISUAL ACUITY IN AFFECTED EYES

The course of visual acuity in affected eyes was defined by the parameters as calculated by the current literature meta-analysis:

$$\mathrm{VA}_t = \mathrm{VA}_0 - \frac{1}{a+\frac{1}{b}}$$

Where

VA_t	= *visual acuity at time-point t;*
VA_0	= *visual acuity at baseline (85 ETDRS Letters);*
a	= *parameter a from meta-analysis = (0.567)*
b	= *parameter b from meta-analysis = (0.0077)*
t	= *time (months)*

One of the characteristics of a discrete-event simulation is that any defined outcome is not continuously updated, but only when an event takes place. This is not an issue as long as there are frequent events and/or the trend of the outcome is linear. As no treatments or other events such as follow-up visits were active in the current simplified model, and the course of visual acuity was not linear, artificial monthly

'update' events were built into the model. This ensured that every month in the simulation, visual acuity and quality of life were updated and traced with precision.

COURSE OF VISUAL ACUITY IN NON-AFFECTED EYES

Visual acuity in non-affected eyes was assumed to remain unchanged over time.

CONVERSION OF NON-AFFECTED TO AFFECTED EYES

Non-affected fellow eyes could progress to active nAMD. In this a discrete-event simulation, this conversion was handled as an event. The timing of this event was defined for the entity at t=0. Using evidence from the literature, we set the simulation to draw a specific time to conversion for each entity, using the hazard rate.

For calculating a hazard rate, we used the survival function:

$$P = 1 - S = 1 - e^{-h \cdot t}$$

Where

P = cumulative incidence of nAMD in the fellow eye;

S = conversion free survival;

t = time (months);

h = hazard rate;

Thus, the hazard rate *h,* which is an instantaneous incidence rate, is defined as:

$$\frac{\ln(1-P)}{t} = h$$

An estimate of *P* was extracted from a natural history review by Wong *et al.*[6] We selected this study as it provides the estimate of incidence in the fellow eye based on the largest base of studies. This review aggregated the results of five studies with 541 patients and found a cumulative incidence of nAMD in the fellow eye *P* of 0.212, at *t* = 36 months.

Assuming that the time-to-event takes on an exponential distribution,[7] the time-to-conversion for the invidivual entity t_i can be defined as:

$$t_i = \frac{-\ln[1-\alpha]}{h}$$

Where α is drawn from a uniform distribution (limits 0; 1)

CALCULATION OF QUALITY OF LIFE

In the simulation, quality of life (QoL) is re-calculated at every event, based on the visual acuity of the better-seeing eye (VA_b) and the parameters from the linear regression analysis from the quality of life study:

$$\text{QoL} = c + d * VA_b$$

Where

c = the constant from the linear regression (0.270)

d = the linear regression coefficient (0.004)

VA_b = visual acuity in the better-seeing eye

REFERENCES IN THE APPENDIX

1. Radner W, Willinger U, Obermayer W, Mudrich C, Velikay-Parel M, Eisenwort B. [A new reading chart for simultaneous determination of reading vision and reading speed]. *Klin Monbl Augenheilkd* 1998;213:174-181.

2. Maaijwee KJ, Meulendijks CF, Radner W, van Meurs JC, Hoyng CB. [The Dutch version of the Radner Reading Chart for assessing vision function]. *Ned Tijdschr Geneeskd* 2007;151:2494-2497.

3. Charlson M, Pompei P, Ales K, MacKenzie C. A new method of classifying prognostic comorbidity in longitudinal studies: development and validation. *J Chronic Dis* 1987;40:373-383.

4. Elshout M, van der Reis MI, Webers CA, Schouten JS. The cost-utility of aflibercept for the treatment of age-related macular degeneration compared to bevacizumab and ranibizumab and the influence of model parameters. *Graefes Arch Clin Exp Ophthalmol* 2014.

5. van Gestel A, Severens JL, Webers CA, Beckers HJ, Jansonius NM, Schouten JS. Modeling complex treatment strategies: construction and validation of a discrete event simulation model for glaucoma. *Value Health* 2010;13:358-367.

6. Wong TY, Chakravarthy U, Klein R, et al. The natural history and prognosis of neovascular age-related macular degeneration: a systematic review of the literature and meta-analysis. *Ophthalmology* 2008;115:116-126.

7. Willekens F. Continuous-time Microsimulation in Longitudinal Analysis. In: Zaidi AH, A. Williamson, P. (ed), *New Frontiers in Microsimulation Modelling.* Vienna, Ashgate; 2009.
